# Effective Learning Support Towards Sustainable Student Learning and Well-Being Influenced by Global Pandemic of COVID-19: A Comparison Between Mainland China and Taiwanese Students

**DOI:** 10.3389/fpsyg.2021.561289

**Published:** 2021-06-22

**Authors:** Ping Xu, Michael Yao-Ping Peng, Muhammad Khalid Anser

**Affiliations:** ^1^School of Teacher Education, Shanwei Polytechnic, Shanwei, China; ^2^School of Economics and Management, Foshan University, Foshan, China; ^3^Business School, Yango University, Fuzhou, China; ^4^School of Public Administration, Xi’an University of Architecture and Technology, Xi’an, China

**Keywords:** social capital, learning support, self-efficacy, student employability, subjective well-being

## Abstract

The novel coronavirus disease that emerged at the end of 2019 began threatening the health and lives of millions of people after a few weeks. However, social and educational problems derived from COVID-19 have changed the development of individuals and the whole country. This study examined the learning method of Taiwanese versus mainland China college students, and evaluated the relationship between learning support mechanism and subjective well-being from a social cognition theory perspective. In this study, a total of 646 Taiwanese questionnaires and 537 mainland China questionnaires were collected to compare the two sample groups in development of students’ subjective well-being. The results showed that social capital and learning support had significant positive correlations with self-efficacy, student employability and well-being and self-efficacy and student employability had significant positive correlations with well-being in Taiwanese sample. In mainland China sample, except paths among social capital, learning support, student employability and well-being, all paths were significant and positive related. Finally, based on the conclusions this study proposed some suggestions specific to theoretical mode for future study.

## Introduction

The influence of learning environment and learning mode of students on learning motivation and learning outcome has always been a key focus in the field of educational psychology (Fantuzzo et al., 2014; Bailey and Phillips, 2016; Jelas et al., 2016). Many studies have found that a good learning environment will help students strengthen their learning motivation and acquire knowledge and skills they need, thus improving the psychological state of achieving goals they set (Bailey and Phillips, 2016; Hanson et al., 2016; Denovan and Macaskill, 2017). Most of these studies were conducted in a complete learning environment. In particular, a majority of these studies have verified the importance of online learning or technology learning (Cheng et al., 2011). However, since the global pandemic of COVID-19 from January 2020, countries all over the world have begun to stop exchanges, such as economics, tourism, and productions, especially educational activities. In order to contain the spread of COVID-19, countries have to cease many economic and educational activities, and postpone the school opening date. In order to enable students to continue learning in the process of combating the epidemic, teachers start to teach students online, which allows students to acquire knowledge with the help of technological carriers. Nevertheless, the impact of teachers’ lesson preparation and students’ acceptance of online learning within a limited period of time on learning outcome remains to be observed (Kramarski et al., 2010), especially because everyone in such a learning context feel anxious and stressed (Bewick et al., 2010; Fantuzzo et al., 2014; Denovan and Macaskill, 2017). Therefore, this study intends to explore the current development status of student learning activities in the context of global pandemic of COVID-19.

A majority of studies on higher education have discussed factors influencing learning outcomes of students (Pike et al., 2012; Bailey and Phillips, 2016), or the application effect of learning models (Pascarella et al., 2013; Campbell and Cabrera, 2014). Some studies in recent years began to discuss the shape of student subjective well-being (SWB) from the view of educational psychology. The emergence of positive psychology leads the psychology (Seligman and Csikszentmihalyi, 2000) into a new direction. Under the influence of the positive psychology, counseling and psychotherapy begin to turn their attention to positive affect subject (Stallman et al., 2018). Many scholars advocate to emphasize the well-being of adolescent, and believe that SWB is the core of adolescent’s mentally healthy development (Miller et al., 2008; Graham et al., 2016). This study replaces student learning outcome with student SWB as the core view: (1) SWB, as the major concerns of student personality and social psychology, is used to examine social change and improvement of educational policies and solve student learning problems (Bailey and Phillips, 2016; Hanson et al., 2016; Stallman et al., 2018); (2) the discussion of student SWB will put emphasis on finding symptoms such as possible depression, anxiety, and psychological disorder (Bailey and Phillips, 2016); the positive and negative psychology lies between two extremes of continuous psychological states, and the more well-being of students will help students face challenges with a positive psychological state, and increase the value of learning course (Stallman et al., 2018). Considering the above reasons, this study aims to further understand and discuss the development course of student SWB through enhancing student employability (SE) in the learning process. (3) From the angle of cross-culture, it can be seen that there are same measurements for learning outcome in different cultures, But in terms of SWB, western culture upholds individual feeling and independence, while oriental culture puts emphasis on social norms and the value of sharing and co-fusion. Western and oriental cultures also have varied ways of understanding, experiencing and pursuing well-being. Based on the above reasons, this study aims to explore the development of students’ SWB in the changing learning activities.

The social cognitive theory contributes to building an appropriate research framework to discuss the relevance between learning activities, environmental influencing factors and psychological needs (Cupani et al., 2010; Bocanegra et al., 2016; Burga et al., 2020). According to the social cognitive theory (SCT), Bandura (1986) hold that personal attribution, environmental influencing factors and intentional behaviors will form a triangular relationship of interaction (Lent et al., 2017; Lent et al., 2018). In other words, individual behaviors are formed by the interaction of individual’s inner thoughts, emotions and environment (Jelas et al., 2016). It is found from the SCT architectural pattern that there is an indirect effect of personal cognitive factors between environmental factors and behavioral factors. In other words, when personal cognitive factors are expected to directly affect student SWB (Lent et al., 2017, 2018), the effect of external environmental factors on student SWB becomes negligible (Fantuzzo et al., 2014; Hanson et al., 2016; Jelas et al., 2016). Self-efficacy is not only the belief of students in their own successful performance and specific behaviors and abilities related to education (Van Dinther et al., 2011), but also an important factor inspiring spontaneous learning motivation and engagement (Parker et al., 2006; Lent et al., 2017, 2018), as well as the core of SCT. Thus, this study proposes that the combination of cognitive factors and the social cognitive theory between self-efficacy and student SWB is supposed to enrich the existing literature.

Moreover, in the aspect of individual cognitive factors, the Pygmalion effect in educational psychology stresses that the external links of students will influence learning intentions and learning outcomes of students. When students perceive expectation and affirmation of important others, they will perform better (Kramarski and Michalsky, 2010; Holfve-Sabel, 2014; Bailey and Phillips, 2016; Graham et al., 2016; Stallman et al., 2018). Scholars have found that the interaction of students with important others such as families, teachers and peers will have an effect on their learning interests and learning outcomes (Bojuwoye et al., 2014; Bailey and Phillips, 2016; Hanson et al., 2016). Further observations show that students have more perseverance and confidence in face of learning challenges if they obtain more kindness, care and support from important others (Kramarski et al., 2010; Jelas et al., 2016; Wibrowski et al., 2017). This study proposes that social capital and learning support are important external environmental cognitive factors in the process of student learning, and employability is the learning output (i.e., skills and knowledge). Regarding the psychological and sociological characteristics, this study is based on students’ social capital (Pike et al., 2012; Peng, 2019) and learning support (Cheng et al., 2011; Bojuwoye et al., 2014; Bailey and Phillips, 2016). The connections of teacher, family, and peer influence students’ employability, changing their cognition of study and assignments. The concept of social capital and learning support pertains to the resources (Cheng et al., 2011; Bojuwoye et al., 2014; Holfve-Sabel, 2014). Social capital and learning support are the most important resources for students to gain more self-efficacy and enhance their employability (Kramarski et al., 2010; Van Dinther et al., 2011; Jelas et al., 2016).

According to the report of Bloomberg in March 2020, only students in Taiwan and Sweden attend the school as usual due to the global pandemic of COVID-19. In order to explore the differences of regions in learning activities caused by environmental threat factors and the changes of student SWB (Lent et al., 2017, 2018), students in Mainland China and Taiwan were taken as the research samples of the interregional comparison in order to learn about the relevance of the research variables. Therefore, this study focuses on determining university students’ perceptions of the psychological and sociological drivers of employability, self-efficacy and well-being in higher education, as well as the relationships among them. The following questions are investigated:

(1) Are there significant associations among students’ perceptions of social capital, learning support, self-efficacy, employability and well-being?
(2) Do students’ employability and self-efficacy play mediating roles in the relationship between the antecedents (psychological and sociological drivers) and consequences of well-being?
(3) Due to Global Pandemic of COVID-19, do various learning activities influence the effect of students’ learning antecedents on self-reported gains in well-being?

## Literature Review and Hypotheses Development

### Theoretical Background of Social Cognition Theory

Social Cognitive Theory (SCT) as an initial foundation in this study for effective learning support toward sustainable student learning and well-being Influenced (Bocanegra et al., 2016; Lent et al., 2017, 2018). SCT is an empirically validated model that have been widely accepted (Cupani et al., 2010; Burga et al., 2020). It is a method for understanding and predicting changes in human behaviors and cognitive behaviors. According to this theory, human meta-development occurs through continuous interaction with the external environment, and the environment must go through a cognitive process before affecting human behaviors (Bocanegra et al., 2016; Lent et al., 2017, 2018). The theory proposes that there is a ternary interactive and causal relationship between cognitive factors, environmental factors and human behaviors. Behavior is influenced by both cognitive and environmental factors (Bandura and Wood, 1989; Van Dinther et al., 2011). Specifically, cognitive factors refer to individual’s cognition, emotion and actual events, and environmental factors refer to the social and physical environments that can affect human behaviors (Cupani et al., 2010; Burga et al., 2020).

According to Bandura, self-efficacy is the key structure of SCT and is believed to have a direct impact on behavior (Cupani et al., 2010; Bocanegra et al., 2016). The outcome expectation is the second structure of SCT, representing a person’s judgment on the consequences resulting from the execution or non-execution of a specific behavior (Bandura, 2004). The pattern of manifestation of outcome expectation can be embodied as self perception (Bandura, 1997), such as SWB. The goal is the third core structure of SCT, and can have a direct impact on behavior and regulate other structures in the model (Bandura, 2004). Achievement of goals requires specific self-regulation skills, such as gaining employability and completing specific goals.

Although Bandura clearly described a social cognitive structural network (Bandura and Wood, 1989), self-efficacy in the past studies has received more attention than other model groups (Van Dinther et al., 2011), or only one or two other variables are used to examine the self-efficacy (Rhodes and Nigg, 2011). This study believes that self-efficacy cannot be studied in isolation. We will use the SCT framework to further understand the impact of changes in the learning environment of students in mainland China and Taiwan during the global epidemic of COVID-19 on SWB. More specifically, the purpose of this study is to examine the impact of social capital (Pike et al., 2012; Peng, 2019) and learning support on self-efficacy and employability, analyze the relationship with student SWBs, and determine whether the effect arising from such relationship varies with regions.

### Subjective Well-Being

People will eventually begin to reflect on the self-seeking of material satisfaction, further seeking psychological satisfaction and beginning to emphasize the importance of quality of life (Lent et al., 2017, 2018); thus the proposal of the concept of SWB (Hanson et al., 2016; Denovan and Macaskill, 2017; Stallman et al., 2018). SWB is a result of satisfaction of life coupled with perceived positive and negative emotional intensity (Evans et al., 2017). Keyes and Waterman (2003) and Keyes (2005) expanded the definition to incorporate the concept of “social well-being” by merging the two (psychological well-being and emotional well-being) to delineate SWB as a sum of three aspects: in the sense of psychological well-being, it serves to explore self-psychological adjustment and the macro-consciousness of the individual’s inner self; a sense of evaluating the function of the self in life through public and social norms; and lastly, emotional well-being as the individual’s awareness and assessment of the emotional state of self-life (Evans et al., 2017).

For a long time, students in higher education have been facing many psychological and physical pressures that make students fail to handle learning challenges with a positive attitude (Bailey and Phillips, 2016; Stallman et al., 2018). Bewick et al. (2010) point out, in a study taking British students as the research object, that college students often have considerable pressure on loan, life and scholarship applications compared with their non-student peers (Bailey and Phillips, 2016; Denovan and Macaskill, 2017), and emphasize that scholars should shift their focus from learning outcome to the discussion of psychological problems of students (Hanson et al., 2016). Although scholars have discussed student SWB from different levels, there are still some research gaps that are worth discussing and exploring (Bailey and Phillips, 2016; Lent et al., 2017, 2018; Stallman et al., 2018), such as how SWB develops, and internal and external factors that affect students’ mental health and SWB (Fantuzzo et al., 2014; Graham et al., 2016; Stallman et al., 2018). In addition, Folkman and Moskowitz (2000) point out in their research that the future research should focus on the discussion of positive emotions and SWB, because it is impossible to find relevant factors that can effectively reduce mental health problems derived from stress if it is not discussed from the perspective of positive outcomes (Hanson et al., 2016; Denovan and Macaskill, 2017). Therefore, based on the social cognitive theory, this study uses SWB as the outcome variable to explore the influence of relevant factors on it. Understanding different mechanisms that contribute to students’ overall well-being is of interest to parents, faculty, staff, and administrators (Hanson et al., 2016; Stallman et al., 2018).

### Developing Subjective Well-Being in Higher Education

Two causal mechanisms contribute to SWB development in higher education: social capital (Pike et al., 2012; Peng, 2019) and learning support (Cheng et al., 2011). Given well-being building support, institutions or faculties can devise the learning context, such as psychological and essential factors, to enhance efficiency and responsiveness of knowledge learning (Wibrowski et al., 2017). Scholars claim that institutions or faculties utilize, integrate, and reconfigure internal and external factor to building an optimal learning context for constructing students’ SWB (Van Dinther et al., 2011). Institutions or faculties implement series of support activities to pinpoint internal and external factor, where social capital focuses on sensing external market information (Pike et al., 2012; Peng, 2019) and learning support on acquiring, assimilating, transforming, and applying learning knowledge (Cheng et al., 2011; Wibrowski et al., 2017). This study considers a better way to build SWB as facilitating adaptation of support activities for social capital and learning support.

#### Well-Being Building Support Mechanism: Social Capital

Social capital is defined as “the aggregate of the actual potential resources which are linked to possession of a durable network of more or less institutionalized relationships of mutual acquaintance and recognition” (Bourdieu and Richardson, 1986). According to Nahapiet and Ghoshal (1998), social capital is the existing or latent embedded resources acquired or transferred by individuals or social entities from social relationships. Social capital in universities is assumed by Peng (2019) to include community engagement, peer relationships, and relations between students and teachers. Social capital can be considered as a significant inherent supporting factor in the learning process. Stallman et al. (2018) indicate that the mental development of students may be negatively affected by isolation, while their learning and competences will benefit from good social support. In the process of interactions in social networks, students maintain their interest, learning motivation, attitude and efforts, which make them realize that they are accountable for their own future. Social capital enables students with higher socio-economic status to develop better relationships with teachers and peers, thus forming rich social capital that will create advantages or them in the social stratum (Pike et al., 2012; Peng, 2019). Pike et al. (2012) mention that students who get along well with their teachers and adapt well to the environment would achieve better academic performance.

Students are facing changes in both learning and living environments, so the social capital is divided into the social capital in terms of learning and the social capital in terms of life. Through the adjustment of social capitals with different structure (Pike et al., 2012; Peng, 2019), students can obtain inner support and encourage required, or acquire resources and information required for changing learning modes, thus enhancing the confidence in completing learning tasks (Stallman et al., 2018). Moreover, the high degree of social capital represents the common language, values, or goals between students and others (Van Dinther et al., 2011). For instance, when the peers have a closer relationship, they would exchange more accurate information. In this case, students are more willing to devote necessary efforts to learning tasks, and successfully finish challenging schoolwork. Therefore, this study proposes H1:

H1: Social capital has a positive and significant impact on students’ self-efficacy.

Students’ social capital also includes the relation across communities, and the links formed based on common interests. For example, peer or communities in schools are a horizontal linking mechanism, which is conducive to links to external resources and information exchange, and facilitates the connection and interaction between heterogeneous populations or communities. Read et al. (2009) indicate that the richness and diversity of social capital in terms of knowledge and information transfer contents will enable students to overcome inefficiencies in learning skills, absorb skills and knowledge required for employment, and facilitate students to enrich themselves (Bauernschuster et al., 2010). Social capital has two direct interests: information and influence. It does not only accelerate the obtainment of information (Shane and Venkataraman, 2000; Van Dinther et al., 2011), but also improves information relevance and information quality (Adler and Kwon, 2002; Burt, 2009). For instance, individuals have close connections with colleges and universities, so they may get in touch with researchers through alumni to get emerging technology information that is to be commercialized. Therefore, this study proposes H2:

H2: Social capital has a positive and significant impact on student employability.

Adler and Kwon (2002) think that individuals can possess or increase beneficial social capitals through specific relational structure and interpersonal interaction, thus achieving individual goals and enhancing the personal sense of achievement. Szreter and Woolcock (2004) mentioned the importance of social network and support for SWB. In other words, in the learning process, students maintaining a good relationship with friends, peers, and teachers, or who is able to obtain proper helps from them, have higher social capital. This is conducive to improving personal feelings of SWB. In the learning process, the degree of mutual assistance among schoolmates will affect learning satisfaction and learning efficiency. Thus, more social capitals have a positive effect on the improvement of positive emotions (Helliwell and Huang, 2010; Helliwell et al., 2014). Likewise, students can mitigate the influence caused by bad environmental events using these accumulated social resources in face of negative environmental events or in need of assistance. When students feel greatly stressed, and importance resources are losing, students’ estimation on stress scenario will be affected if they have enough social resources, thus reducing adaptive strategies for negative emotions and improper use (Nohe and Sonntag, 2014). Therefore, this study proposes H3:

H3: Social capital has a positive and significant impact on students’ SWB.

#### Well-Being Building Support Mechanism: Learning Support

Key classroom learning experience plays an important role in the learning process, and diversified social and academic integration activities happen in class, which make classroom learning experience a concern in the structure of higher education (Demaris and Kritsonis, 2008; Cheng et al., 2011; Jelas et al., 2016). In order to allow students to feel the enriched classroom learning experience and get learning skills, the explicit and essential learning support provided by relevant elements in class (Cheng et al., 2011; Jelas et al., 2016) will facilitate students to form norms for joint compliance from informal activities, strengthen cooperative behaviors among students and improve their problem-solving capability. According to Mashau et al. (2008) learning support includes supplementary, curriculum advice, academic mentoring, remedial or extra class instructions, assisting students to work in groups, developing study and note-taking skills, academic mentoring, school psychological services, medical and social work services, feeding scheme, and all other services for meeting special needs of learners and for preventing learning difficulties (Kramarski et al., 2010; Cheng et al., 2011). Chang et al. (2013) indicate that social relationship with peers and teachers is able to form a positive learning atmosphere, including the supportiveness from teacher traits and peer traits, thus facilitating students to obtain good learning experience. Bojuwoye et al. (2014) state that effective learning supports can maximize gains from available teaching and learning activities, enabling students to overcome learning disabilities, and enhance their esteem, acceptable social behaviors and academic success (Mashau et al., 2008; Wibrowski et al., 2017). By reference to the study of Chang et al. (2013), this study identifies with the opinion that learning support includes teacher supportiveness and peer supportiveness, which are used to measure this dimension.

Teacher supportiveness is the most direct and effective knowledge source for students. Teachers would assist students in school demands, accept the application of different courses, and solve confusions and anxiety arising from the application of technological learning in learning (Cheng et al., 2011; Bojuwoye et al., 2014). Besides, the support for effective learning through teaching innovation will improve the status of learning engagement (Jelas et al., 2016), intensify learning motivation and perfect the learners’ successful learning scenarios (Kramarski et al., 2010). Learning support is also related to theories of learning motivation (Wibrowski et al., 2017). Combined with psychological features of students, conducive learning environments can be created to enable students to be more confident in completing schoolwork (Mulholland and O’Connor, 2016). Students will be more driven and motivated to engage in learning and understand values and insights brought by learning, thus improving student self-efficacy, if they feel the positive psychological environment established by learning support from teachers and peers. Therefore, this study proposes H4:

H4: Learning support has a positive and significant impact on students’ self-efficacy.

Moreover, the learning support, with its relationship with SE, is helpful in improving students’ interest in learning and the application of their professional skills, and in further enhancing students’ capability (Cheng et al., 2011; Jelas et al., 2016). When facing practical problems, such as critical analysis, problem solving (Kramarski et al., 2010), and reflection, students can demonstrate better learning attitudes and critical thinking ability. Mulholland and O’Connor (2016) have confirmed that students who have accepted the learning support pattern will change their learning motives, attitudes, and behaviors so as to enhance their critical thinking, learning autonomy, and employment-related competencies (Wibrowski et al., 2017). Therefore, this study proposes H5:

H5: Learning support has a positive and significant impact on SE.

By strengthening psychological characteristics of student, learning support promotes better schoolwork engagement and effective learning (Gasiewski et al., 2012; Jelas et al., 2016). Scholars have found that learning engagement and learning motivation are often restricted by sense of learning incapability and sense of learning helplessness caused by learning disabilities in the learning process (Bewick et al., 2010; Cheng et al., 2011; Denovan and Macaskill, 2017). Thus, the emphasis of higher education lies in intensifying the positive psychological factors of students. Under the learning support from teachers and peers, the strong social relationship will improve psychological characteristics of students (Jelas et al., 2016), enabling students to recognize their own responsibilities and satisfy their needs for society and affection. Furthermore, if students are able to obtain support that is not provided for external students, they will be more willing to spend more time and devote more energy to learning, thus generating positive attitudes and SWB. Therefore, this study proposes H6:

H6: Learning support has a positive and significant impact on students’ SWB.

### Student Employability (SE)

In recent years, scholars have put more effort into employability-related research. The substantial technological, social, and economic changes that have occurred in recent decades (Abbas et al., 2015) have modified the concepts and operations of industrial organizations (Abbas and Sağsan, 2019) and HEIs across the world (Vermeulen et al., 2018). Hence, dynamic HEIs ensure the highest standards of human capital development, so that they can contribute to economic growth (Ahmed et al., 2015; Baek and Cho, 2018). Through research situations and design of methods, and the integration of theoretical and practical analysis, scholars have studied the meaning of employability and the causality between employability and other factors (Hennemann and Liefner, 2010; Avramenko, 2012; Baek and Cho, 2018). Van Der Heijde and Van Der Heijden (2006) have argued that employability is the individual’s appropriate application of competence (Blázquez et al., 2018), continuous acquisition and creation of essential work skills in order to accomplish all the tasks, and adaptation to internal and external labor market changes (De Cuyper et al., 2008; Vermeulen et al., 2018; Malik et al., 2019). Hence, the need for critical and reflective thinking, problem-solving abilities, self-management, learning, and related competencies is continually increasing across all disciplines (Makkonen and Olkkonen, 2017). Several prior studies have indicated that in addition to the influence of basic education on employability, factors like personal conditions, interpersonal relations, and external factors that cannot be acquired in higher education should also be considered (Ahmed et al., 2015; Cacciolatti et al., 2017; Blázquez et al., 2018).

Hennemann and Liefner (2010), who developed a graduate employability training process, summed up a comprehensive structure of impact factors to explain the capacity, capability, and competence (Blázquez et al., 2018) that are important elements in the process of developing employability (Hennemann and Liefner, 2010; Modestino, 2016; Lurie and Garrett, 2017; Blázquez et al., 2018; Likisa, 2018). De Cuyper et al. (2008) considers employability as having its importance in the post-industrial knowledge society by continuously updating knowledge to maintain competitiveness in a global market (Griffeth et al., 2005), and making them feel capable of dealing with temporary and future developments—new psychological contracts created by individuals will likely increase their well-being. In addition, individuals can process the same things and tasks more efficiently and in less time with relevant experience, updated skills and knowledge—as well as a well-developed social network—so as to improve employability (Griffeth et al., 2005). The abundance of time saved will be used for life needs and personal future planning, thereby enhancing happiness. Similarly, students with higher employability can face the challenges of the future with a broader perspective. In addition to mastering the content of school work, they also have a more precise direction for planning and preparation for entering the workplace, reducing their insecurity and enhancing SWB. Based on the above phenomena, the hypothesis of this study is as follows:

*H7: SE has a positive and significant impact on students’ SWB.*

### Self-Efficacy

Social cognition scholars argue that individuals’ behavioral outcomes will be influenced by both environmental and cognitive factors in a given situation (Van Dinther et al., 2011), especially those beliefs that lead to success and behavior (Wang et al., 2016; Lent et al., 2014). They call these beliefs “self-efficacy,” an important cognitive variable in personal factors during the process of interpreting individual formative behaviors, and interaction with the environment (Lent et al., 2014; Sheu et al., 2014). It can also be seen as the basis for human behavioral motivation, mental health and personal achievement (Dacre Pool and Qualter, 2013). Self-efficacy is widely used in the field of education to explore the psychological cognitive factors of students of different ages and their positive impact on academic achievement and student career development (Wang et al., 2016).

According to the above discussion, students who have confidence in their abilities will have more efficient behavior and better interpersonal relationships than those who do not. According to Dacre Pool and Qualter (2013), highly self-motivated students look for resources and opportunities to accomplish tasks that exist in social networks (Van Dinther et al., 2011). Only by establishing and maintaining network relationships can they achieve their goals. Knowledge and resources are needed (Lent et al., 2014; Sheu et al., 2014). Furthermore, teamwork can also be seen as a strong network relationship, and the process of students solving problems and achieving tasks through teamwork will positively affect their employability. It is pointed out that, according to the above, this study proposes the following H2:

*H8: Self-efficacy has a positive and significant impact on SE.*

Some scholars have focused their investigations on mental health concerns, social support, and coping styles in low SES college students (Song and Ingram, 2002; Tong and Song, 2004). However, few studies thus far have tapped this population’s general self-efficacy and SWB (Evans et al., 2017). Tong and Song’s (2004) research findings indicated that low-SES college students reported a lower level of social support, limited sources of support, and low perceived support (Song and Ingram, 2002). It implies that low-SES college students’ general self-efficacy and SWB decrease because they are unable to receive timely and necessary psychological support when confronting stress. In addition, it might contribute to unique stressors. Conversely, students with higher self-efficacy have higher SWB (Evans et al., 2017). In summary, the study infers the following:

*H9: Self-efficacy has a positive and significant impact on students’ SWB.*

**FIGURE 1:**
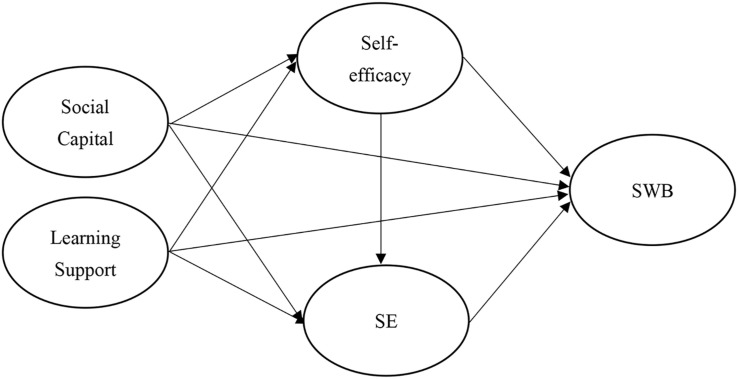
Research framework.

Based on the above hypotheses, this study proposes the following research framework Figure 1.

## Methodology

### Sampling

The research sample in this study comprised undergraduates. Purposive sampling was adopted, since there are many mathematics departments in universities, and different universities have different theories on school management and different teaching characteristics. To understand whether the subject attributes would influence the research results, the different research variables between students majoring in natural sciences and those majoring in social sciences were verified. The results indicated that subject did not significantly impact the research variables, so did not need to be included as an independent variable in subsequent analyses. This study proposed a framework to explore the correlations and development mode of social capital, learning support, self-efficacy, SE and SWB. It sampled from Taiwanese and mainland China universities. This study also incorporates students’ degree as a sampling condition, as freshmen were left out of sample. This study selected 12 Taiwanese universities and 6 mainland China universities, and then sent 2,000 questionnaires to each of them. The researchers contacted with the colleges and teachers who were willing to receive the questionnaire by telephone and email first. The survey packages were sent by post to students of 18 universities. Each survey package contained a covering letter explaining the survey purpose, a survey instrument and a postage-paid envelope. Before filling out the questionnaires, students have been asked to understand the right of attending survey to ensure research ethical aspects. The students voluntarily completed the questionnaires, after signing their informed consent. During the school year (2020.03–2020.04), students completed the questionnaire. After sampling, a total of 646 Taiwanese questionnaires and 537 mainland China questionnaires were returned, for an effective response rate of 64.6 and 53.7%. Since freshmen were not familiar with the learning process, participants were sophomores, junior and senior students.

### Measures

All constructs were measured by multiple-item scales based on previous studies. The construct of social capital was divided into student–faculty interaction and interpersonal environment. This study adopted the scale proposed by Pike et al. (2012). Student–faculty interaction was measured using four items, and interpersonal environment was measured using three items. The construct of learning support was divided into peer supportiveness (3 items) and teacher supportiveness (3 items). This study adopted the scales proposed by Pike et al. (2011). Similar to the employability scale reported by Pan and Lee (2011), eighteen items were used to capture general ability for work (GAW) (8 items), professional ability for work (PAW) (4 items), attitude at work (AW) (3 items) and career planning and confidence (CPC) (3 items). For self-efficacy, six items were selected on the basis of prior scale and item analyses of Asian applications (Rigotti et al., 2008). Subjective Well-being was measured using Keyes’s (2005) Subjective well-being instrument (adolescent version), which comprehensively assesses well-being in terms of emotional (3 items), psychological (4 items) and social (4 items) dimensions. All items were measured with a five-point Likert scale (1 = totally disagree; 5 = totally agree).

## Results

### Sample Description

After sample collection, backgrounds of Taiwanese and mainland China samples in Table 1 are arranged in this study. Before conducting model verification, we have verified that whether different backgrounds vary in SWB. In this study, the independent samples t-test and one-way analysis of variance were applied to compare differences of gender, part-time job, scholarship, first-generation college student, majors, dedication to class preparation and weekly study hours spent on major courses on students’ SWB in Taiwanese and mainland China samples. Results show that when it comes to weekly study hours spent on major courses, significant differences occur in students’ SWB. Thus, when conducting model verification, sample backgrounds will not be added in this study. Table 1 shows descriptive statistics of Taiwanese and mainland China samples.

**TABLE 1:** Descriptive statistics by Taiwanese and mainland China samples.

Characteristic	Scale	Taiwan		Mainland china	
Gender	Male	310	−0.453 (*p* > 0.1)	329	−1.060 (*p* > 0.1)
	Female	336		208	
Part-time job	Yes	389	1.364 (*p* > 0.1)	343	0.661 (*p* > 0.1)
	No	248		194	
Scholarship	Yes	287	−0.250 (*p* > 0.1)	215	0.492 (*p* > 0.1)
	No	357		322	
First-generation college student	Yes	410	0.052 (*p* > 0.1)	372	0.995 (*p* > 0.1)
	No	236		165	
Majors	Social science	355	−0.137 (*p* > 0.1)	264	1.198 (*p* > 0.1)
	Natural science	291		273	
Dedication to class preparation	Yes	352	−0.899 (*p* > 0.1)	332	−0.773 (*p* > 0.1)
	No	294		205	
Weekly study hours spent on major courses	Less than 5	421	6.289 (*p* < 0.01)	186	3.434 (*p* < 0.01)
	5 to less than 10	93		136	
	10 to less than 15	52		107	
	15 to less than 20	36		87	
	More than 20	44		21	

### Reliability and Validity

All scales used in this study were found to be reliable, with Cronbach’s α ranging from 0.83 to 0.96. Table 2 shows the reliability of each scale, and the factor loadings for each item therein. In order to gauge validity, this study employed confirmatory factor analysis (CFA) using AMOS 23.0 to verify the construct validity (both convergent and discriminant) of the scales. According to Hair et al. (2006) recommended validity criteria, CFA results show standardized factor loading of higher than 0.7; average variance extracted (AVE) ranges between 0.539 ∼ 0.729; and composite reliability (CR) ranges between 0.800 ∼ 0.918. All three criteria for convergent validity were met, and correlation coefficients were all less than the square root of the AVE within one dimension, suggesting that each dimension in this study had good discriminant validity.

**TABLE 2:** Measurement properties.

		1	2	3	4	5	6	7	8	9	10	11	12
1. Interaction		0.87/0.71	0.53	0.32	0.54	0.00	0.08	0.06	0.04	0.02	0.01	-0.02	0.00
2. Interpersonal		0.65	0.76/0.77	0.32	0.42	−0.03	0.07	0.05	0.05	0.02	−0.07		
3. Peer		0.34	0.41	0.82/0.72	0.58	0.11	0.12	0.05	0.07	0.01	0.02	−0.08	−0.03
4. Teacher		0.42	0.57	0.63	0.89/0.71	0.13	0.04	0.05	0.08	0.03	0.02	−0.03	−0.03
5. Self-efficacy		0.40	0.49	0.47	0.51	0.79/0.76	0.09	0.11	0.12	0.11	0.28	0.29	0.32
6. GAW		0.44	0.53	0.43	0.51	0.50	0.74/0.71	0.13	0.14	0.10	0.01	0.07	0.07
7. PAW		0.40	0.44	0.41	0.49	0.49	0.70	0.79/0.77	0.62	0.63	0.13	0.08	0.13
8. AW		0.42	0.53	0.51	0.54	0.55	0.71	0.74	0.75/0.73	0.70	0.19	0.13	0.19
9. CPC		0.49	0.49	0.43	0.49	0.56	0.65	0.64	0.73	0.82/0.84	0.16	0.14	0.17
10. Emotional		0.39	0.48	0.41	0.44	0.56	0.40	0.38	0.48	0.44	0.87/0.77	0.74	0.61
11. Psychological		0.45	0.52	0.46	0.54	0.69	0.51	0.48	0.52	0.51	0.79	0.81/0.75	0.71
12. Social		0.53	0.52	0.43	0.48	0.62	0.47	0.44	0.51	0.50	0.68	0.72	0.84/0.77
Mean	Taiwan	3.22	3.56	3.72	3.80	3.75	3.54	3.63	3.61	3.55	3.63	3.71	3.52
	China	3.59	3.23	3.63	3.63	3.95	3.71	3.88	3.93	4.01	4.40	4.47	4.58
SD	Taiwan	0.81	0.70	0.69	0.71	0.63	0.64	0.70	0.69	0.72	0.72	0.69	0.77
	China	0.58	0.72	0.59	0.55	0.40	0.63	0.72	0.69	0.71	0.51	0.49	0.50
α	Taiwan	0.93	0.80	0.86	0.92	0.91	0.90	0.87	0.78	0.86	0.90	0.88	0.90
	China	0.78	0.81	0.68	0.64	0.77	0.75	0.85	0.76	0.88	0.78	0.81	0.85
AVE	Taiwan	0.76	0.58	0.68	0.80	0.62	0.55	0.63	0.57	0.67	0.75	0.65	0.71
	China	0.51	0.59	0.52	0.51	0.58	0.50	0.60	0.53	0.71	0.60	0.57	0.59
CR	Taiwan	0.93	0.81	0.87	0.92	0.91	0.91	0.87	0.80	0.86	0.90	0.88	0.91
	China	0.78	0.81	0.77	0.76	0.84	0.79	0.85	0.77	0.88	0.81	0.83	0.84

### The Structural Model Fit of SEM

Social capital, learning support, SE and SWB are often higher-order constructs in nature, with items measuring them as indirect reflective measures of both second- and first-order factors associated with them, where the social capital, learning support, SE and SWB are umbrella terms for multiple sub-constructs. Social capital is often conceptualized as a two-dimensional construct, learning support as a two-dimensional construct, SE as a four-dimensional construct, SWB as a three-dimensional construct. Five constructs comprised the final model: social capital, learning support, self-efficacy, SE and SWB. This study adopted first-order constructs to assess structural model. Fit indices greater than 0.90 benchmark (GFI = 0.949, AGFI = 0.915, TLI = 0.945, and CFI = 0.963) indicated data fits said model. Similarly, levels of misfit were tolerable, with RMSEA = 0.054 and RMR = 0.033, which RMSEA and RMR were below the relevant benchmark of 0.08. Additional tests included normed chi-square of 2.57 (less than benchmark of 5) and SRMR = 0.035 (less than benchmark of 0.08).

**FIGURE 2:**
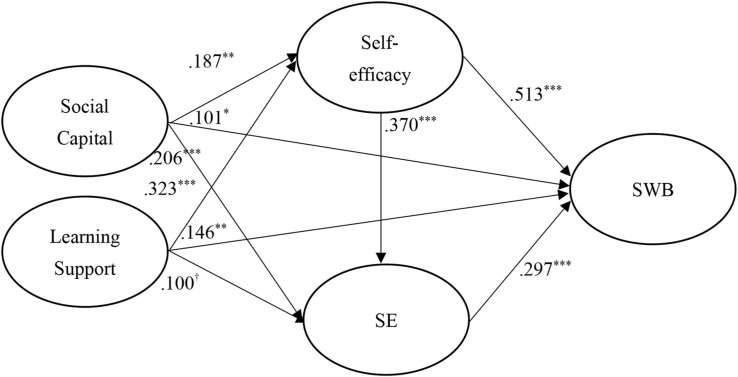
Structural model. ^†^*p* < 0.1; *p < 0.05; **p < 0.01; ***p < 0.001.

Figure 2 shows the results of the hypothesized relationships and standardized coefficients. This study finds that social capital relation has positive effects on self-efficacy (β = 0.187, *p* < 0.01), SE (β = 0.206, *p* < 0.001), and SWB (β = 0.101, *p* < 0.05), the learning support relation would be positively associated with self-efficacy (β = 0.323, *p* < 0.001), SE (β = 0.100, *p* < 0.1) and SWB (β = 0.146, *p* < 0.01), the self-efficacy would be positively associated with SE (β = 0.370, *p* < 0.001) and SWB (β = 0.513, *p* < 0.001), the SE relation has a positive effect on SWB (β = 0.297, *p* < 0.001). Accordingly, H1, H2, H3, H4, H5, H6, H7, H8, and H9 were acceptable and supported.

The model proposed in this study assumed that self-efficacy and SE would mediate the relationship among social capital, learning support and SWB. This study further tests for mediation following the approach proposed by Sobel (1982). In above structural model, mediation results indicated that social capital → self-efficacy → SWB (*Z* = 2.830, two-tailed probability *p* < 0.01), social capital → SE → SWB (*Z* = 3.125, two-tailed probability *p* < 0.01), learning support → self-efficacy → SWB (*Z* = 4.935, two-tailed probability *p* < 0.001), and learning support → SE → SWB (*Z* = 1.776, two-tailed probability *p* < 0.1) were positively significant. Therefore self-efficacy and SE have full mediations on social capital and learning support toward SWB.

### Multiple Group Analysis: Taiwan and Mainland China

It was confirmed that the measurement pattern was stable. However, in order to avoid overgeneralizing the data-driven patterns and theories, the study followed the suggestion of Hair et al. (2010) to divide the sample data into two groups based on regions (646 Taiwanese and 537 mainland China students, respectively). Besides, multiple group testing was combined with bootstrapping to gradually control the pattern parameters of the groups. The nested models developed from the different limitations χ*^2^* difference quantity to make significance analysis, in order to determine the reasonability of those parameters in controlling the two groups. The results are shown in Table 3.

**TABLE 3:** Multi-group testing.

Model	χ*^2^*	*df*	χ*^2^/df*	*p*	RMSEA	NFI	ECVI	0.9 CI
1. Unconstrained	650.156	210	3.096	0.000	0.042	0.946	0.713	(0.713 ∼ 0.781)
2. Measurement weights	889.387	222	4.006	0.000	0.05	0.926	0.895	(0.821 ∼ 0.976)
3. Structural weights	1110.552	229	4.85	0.000	0.057	0.908	1.071	(0.986 ∼ 1.162)
4. Structural covariances	1162.203	232	5.009	0.000	0.058	0.904	1.109	(1.022 ∼ 1.203)
5. Structural residuals	1240.539	235	5.279	0.000	0.06	0.897	1.171	(1.080 ∼ 1.267)
6. Measurement residuals	1764.892	252	7.004	0.000	0.071	0.854	1.586	(1.476 ∼ 1.702)
2–1	239.231	12		0.000		0.020		
3–1	460.396	19		0.000		0.038		
4–1	512.047	22		0.000		0.042		
5–1	590.383	25		0.000		0.049		
6–1	1114.736	42		0.000		0.092		

The analysis results show that the value of each pattern mode of χ*^2^*/*df* ranged from 3.096 to 7.004, the RMSEA ranged between 0.042 and 0.071 and the ECVI was within 90% of the confidence interval. It can be learned from Table 2 that the χ*^2^* values of the weighted measurement model, weighted structure model, covariance structure model and residual structure model reached significant levels, which shows that the models had good between-groups invariance. In addition, the NFI added value of each model was less than 0.05, which is in accordance with the standard recommended by Little (1997). Therefore, the framework and conclusion of this research will present good generalized validity.

**FIGURE 3:**
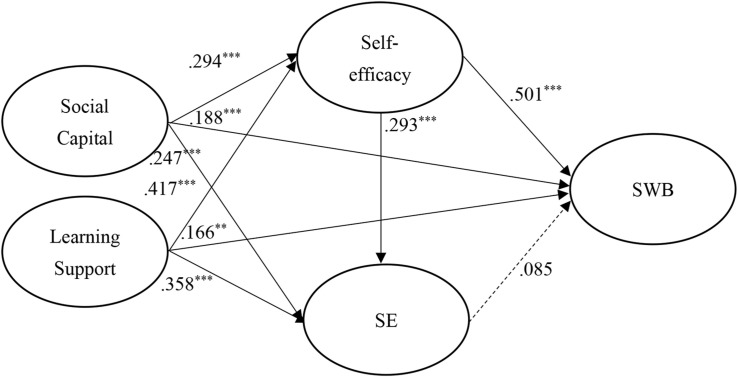
Structural model on Taiwanese students. **p < 0.01; ***p < 0.001.

**FIGURE 4:**
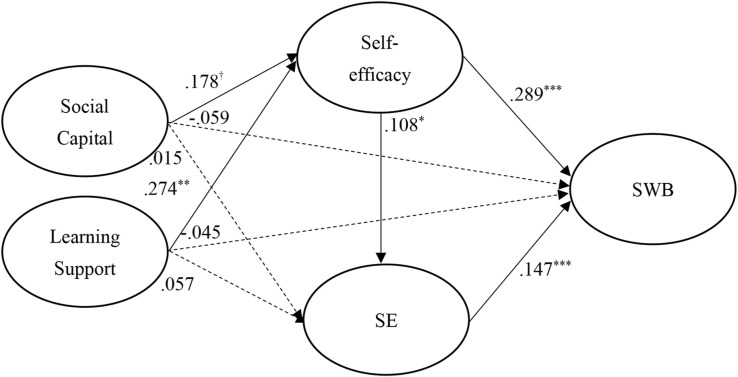
Structural model on mainland China students. ^†^*p* < 0.1; *p < 0.05; **p < 0.01; ***p < 0.001.

The standardized structural weights for Taiwanese and mainland China students are shown in Figures 3, 4, respectively. Specifically, in structural model of Taiwanese students, all paths had significantly positive effects except the effect of SE on SWB. However, comparing to Taiwanese students, in structural model of mainland China students, social capital and learning support appeared to have no significant effects on SE and SWB. This suggests that the Taiwanese students achieved greater SWB development from having well-established social capital and learning support.

However, the results showed in both model that self-efficacy may play a significant mediating role in the relationship among social capital, learning support, SE and SWB. These findings regarding region differences support our study’s purpose with regard to identifying the region-specific pathways to students’ SWB.

## Discussion and Conclusion

This study takes Taiwanese and mainland China students as research samples to test the social capital, learning support, self-efficacy, SE and SWB correlation using the social cognition theory. This study will fill the theoretical gap in the application of Western theories under the Eastern context, and increase the generalization of the theory. Based on our research findings, this study aims to provide following contributions. First, there are few studies to verify students’ learning based on a huge environmental challenge. This study investigates universities students’ learning process and SWB in the situation of Global Pandemic of COVID-19 and attempt to offer practical implications for institutional administrations. Second, most previous studies on SCT explored the importance of environmental factors but merely few studies provided essential contributions with global environmental factors. This study aims to fill the theoretical gap and enrich theoretical foundation of SCT. Third, in addition to verifying the research framework built through SCT in Asian context, this study also includes different perspective of conventional learning (conventional vs. online learning). Our findings will provide more insights and suggestions in terms of learning theories.

The results indicate that the social capital and learning support of Taiwanese students are positively related to their employability, whereas there have no significant effects on mainland China students. These results correspond with those of Cupani et al. (2010); Wu et al. (2010), and Lent et al. (2016); on the basis of SCT, they believe that the learning environment differences between conventional and online learning influence students’ learning status and learning activities, causing knowledge and skills-gaining to differ. Our findings are largely consistent with those of these prior studies, supporting the SCT model’s availability across a range of regions. Besides, there may be insignificant correlations among social capital, learning support and SE on mainland China students because students can not acquire sufficient employment information leaded by economic activity stagnation which fosters suitable employability.

Moreover, the results show positive correlations among social capital, learning support and self-efficacy for both Taiwanese and mainland China students. It is also worth noting that the internal and external learning support mechanism imply that students with more social capital and learning support from peer/teacher are likely to be more involved in the learning environment and actively participate in learning activities, thus obtaining ability and confident of achieving course task, such as the development of systematic/integrative thinking and problem-solving skills. This finding is consistent with the findings of a number of previous studies (Pike et al., 2012; Wu et al., 2012; Bocanegra et al., 2016), supporting the relationship among social capital, learning support and self-efficacy.

Besides, our findings show that social capital and learning support of Taiwanese students are positively related to their SWB, whereas there are no significant effects on mainland China students. Although the results are consistent with argument of Lent et al. (2016) that traits may function along with psychological and essential factors in the maintenance of SWB, limitations of environmental trait may produce differences, such as freedom degree of learning and behavior. A majority of mainland China students learn on scientific media, the learning outcomes are inconsistent with Cheng et al. (2011), and Jelas et al. (2016), i.e., more learning supports fail to result in better SWB. The possible reason may be that Cheng et al. (2011) failed to measure the psychological factors of learners arising from the external environmental threats, and higher emotional, psychological and social well-being cannot be formed despite of higher degree of usage and acceptance of information technology.

Finally, the findings show that self-efficacy and SE are strong contributors to SWB for both Taiwanese and mainland China students. Furthermore, self-efficacy plays a key mediating role in the research model of SCT. These findings are quite consistent with those of Sheu et al. (2014) and Lent et al. (2016), who verified the well-being model cross-sectionally in different samples of college students. Moreover, different from the study of Sheu et al. (2014), this study compares samples of different regions in the same model, such as Taiwanese and Singaporean college students, reports good overall model-data fit in both samples (Taiwan and mainland China), and verifies direct and indirect effects of self-efficacy generated in well-being model of SCT on SWB. However, differing from the studies of Sheu et al. (2014) and Lent et al. (2016), this study also considers psychological effects of global environmental events, and enriches the theoretical model and SCT of well-being based on the region analysis.

### Practical Implications

In sum, according to our findings, this study suggests some important practical implications for improving quality of higher education. Firstly, in this study, the teachers and peers supportiveness and social capital were perceived as equally important and predictive of students’ own perceived levels of self-efficacy, SE and SWB. Internal and external building mechanism of mentality will contribute to students obtaining more resource and psychological supports, which are essential conditions for improving SWB. Thus, at the present stage when countries and regions all over the world combat COVID-19, in face of similar events, institutional administrations should encourage teachers to actively form a close ties with students, build communication platform using technological media and information technology tools, and provide schoolwork or psychological support in real time. Students are afraid and worried about catching the virus, but mainly because they think they can infect their family, specifically in mainland China, and this makes them avoid external contacts in learning. Moreover, the lockdown situation has produced conflicting emotions in the students. On the one hand, they are scared, nervous, lonely, sad, bored and angry, but they also feel safe, calm and happy with their families. These phenomena may use to explain why social capital and learning support were insignificant with SE and SWB.

Second, external environment factors, especially the global epidemic COVID-19, may affect student learning status. Thus, school administrations must be examined for a sense of risk management. On this basis, this study suggests institutional administrations to take preventive risk management measures to tackle with threats and challenges brought by adaptive risks in face of similar events. Although this event causes all students to take online class, not all students are equipped with the required technological media or information technology tools. In consequence, schools should count up the number of students who have information technology tools at first and measure whether courses are able to be taught online; and the courses that are not suitable for online teaching should be adjusted in terms of schedule. On this basis, this study suggests that course should be modified and transferred to the next semester if the online courses have low teaching results or fail to achieve the expected learning outcomes.

Third, in light of the structural patterns of two regions, SWB deriving from self-efficacy of Taiwanese students is superior than that of students in Mainland China. It can be seen that opening schools or not will both have an effect on students. Students in regions that are blocked for longer time tend to feel more helpless, incapable and anxious. The limitations of environmental traits, the difference among learning modes, life modes and interpersonal relationship, etc. caused by COVID-19 would have more effect on their lives and plan, such as the cancelation of GRE, TOEFL, IELTS in February and March will affect their applications for abroad studies in near future, etc. These results indicate the need for Governments to also consider college students in their management of the current situation by placing greater emphasis on social and inclusive policies to help alleviate the possible effects that they may suffer as a consequence of the pandemic and the lockdown.

### Research Limitations

The research results contribute to the literature on region-specific students, SCT, and student well-being; nevertheless, some limitations still exist and represent further research directions. First, social cognitive theory has obtained considerable status in the psychological field, but only a few studies have considered the relationship between building mechanism and well-being of undergraduate students in higher education. Although the building mechanism (social capital and learning support) was constructed with reference to SCT in this study, and important learning theories can be derived from the research results, other motivation theories, such as attribution theory, self-efficacy theory, and hierarchy needs theory, still apply to explain how to trigger learning in region-specific students. Thus, it is suggested that future research can utilize different theoretical models in order to identify relevant psychological dimensions influencing students’ well-being. Second, this study required students to self-report details on their psychological building mechanism as the indicator, mainly because actual data is confidential and not easily obtained. However, errors may exist in the students’ self-statement of their psychological status. The link between building mechanism and well-being may be better understood if students’ actual psychological status is assessed, with due consideration for research ethics. It is an exploratory study to some extent. The SCT model is adopted in the context of COVID-19 to explore the relationship between various variables in the process of student learning, and a comparison of model is further conducted in different regions. Thus, it is difficult to review more previous research results of the same context. For the sake of increasing more theoretical contributions, this study suggests that future researchers can conduct similar model validation in the post COVID-19 period to confirm the changes in the relationship between variables in different situations, so as to provide more abundant insights and implications.

Besides, this study suggests future researchers to include interview contents and students’ observations of learning status in their studies to support the researching results and make a comprehensive judgment. Third, due to restrictions of time and space, only 16 universities were sampled in this study, with 817 valid questionnaires in total. The research objects were divided into Taiwanese and mainland China students. Future research could explore and compare other groups, in addition to expanding the quantity of samples and improving the research representativeness, so as to provide additional insights relevant to higher education policy.

## Data Availability Statement

The raw data supporting the conclusions of this article will be made available by the authors, without undue reservation.

## Ethics Statement

The studies involving human participants were reviewed and approved by Institutional Review Board, University of Taipei. The patients/participants provided their written informed consent to participate in this study.

## Author Contributions

MP and PX contributed to the ideas of research, collection of data, and empirical analysis. MP and PX contributed to the data analysis, design of research methods, and tables. MP and MA participated in developing a research design, writing, and interpreting the analysis. All authors contributed to the literature review and conclusions.

## Conflict of Interest

The authors declare that the research was conducted in the absence of any commercial or financial relationships that could be construed as a potential conflict of interest.
